# 2,3-Diphenyl-2,3-di­hydro-4*H*-1,3-benzo­thia­zin-4-one

**DOI:** 10.1107/S1600536814005881

**Published:** 2014-03-22

**Authors:** Hemant P. Yennawar, Ryan V. Bendinsky, David J. Coyle, Aaron S. Cali, Lee J. Silverberg

**Affiliations:** a Department of Chemistry, Pennsylvania State University, University Park, PA 16802, USA; b Pennsylvania State University, Schuylkill Campus, 200 University Drive, Schuylkill Haven, PA 17972, USA

## Abstract

In the title compound, C_20_H_15_NOS, the dihedral angle between the phenyl rings is 74.25 (6)°. The six-membered 1,3-thia­zine ring has an envelope conformation with the C atom at the 2-position forming the flap. The crystal structure features weak C—H⋯O inter­actions, which lead to the formation of a tape motif along [110].

## Related literature   

For other preparations of the title compound, see: Ponci *et al.* (1963 ▶); Kollenz & Ziegler (1970 ▶); Oae & Numata (1974 ▶); Badea *et al.* (1998 ▶). For previously published methods for the preparation of 1,3-thiazin-4-ones by condensation of an imine with a thio­acid, see: Kamel *et al.* (2010 ▶); Zarghi *et al.* (2009 ▶); Zhou *et al.* (2008 ▶); Srivastava *et al.* (2002 ▶). For the synthesis and crystal structures of related compounds, see: Yennawar & Silverberg (2013 ▶, 2014 ▶); Yennawar *et al.* (2013 ▶).
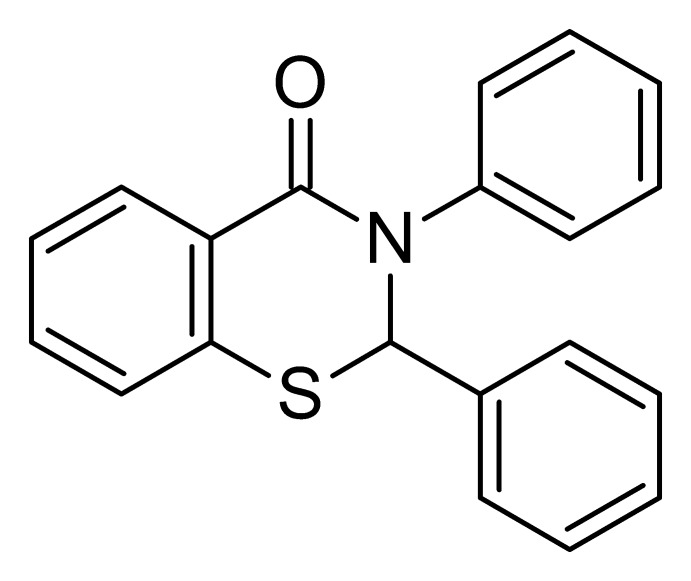


## Experimental   

### 

#### Crystal data  

C_20_H_15_NOS
*M* *_r_* = 317.39
Monoclinic, 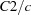

*a* = 14.799 (4) Å
*b* = 9.606 (3) Å
*c* = 22.492 (6) Å
β = 98.736 (5)°
*V* = 3160.1 (15) Å^3^
*Z* = 8
Mo *K*α radiation
μ = 0.21 mm^−1^
*T* = 298 K
0.18 × 0.16 × 0.05 mm

#### Data collection  

Bruker SMART APEX CCD area-detector diffractometer
Absorption correction: multi-scan (*SADABS*; Bruker, 2001 ▶) *T*_min_ = 0.963, *T*_max_ = 0.990
14747 measured reflections
3956 independent reflections
3300 reflections with *I* > 2σ(*I*)
*R*_int_ = 0.021

#### Refinement  

*R*[*F* ^2^ > 2σ(*F* ^2^)] = 0.051
*wR*(*F* ^2^) = 0.127
*S* = 1.06
3956 reflections
208 parameters
H-atom parameters not refined
Δρ_max_ = 0.28 e Å^−3^
Δρ_min_ = −0.25 e Å^−3^

### 

Data collection: *SMART* (Bruker, 2001 ▶); cell refinement: *SAINT* (Bruker, 2001 ▶); data reduction: *SAINT*; program(s) used to solve structure: *SHELXS97* (Sheldrick, 2008) ▶; program(s) used to refine structure: *SHELXL97* (Sheldrick, 2008) ▶; molecular graphics: *XSHELL* in *SHELXTL* (Sheldrick, 2008) ▶; software used to prepare material for publication: *ORTEP-3 for Windows* (Farrugia, 2012 ▶).

## Supplementary Material

Crystal structure: contains datablock(s) I. DOI: 10.1107/S1600536814005881/fy2112sup1.cif

Structure factors: contains datablock(s) I. DOI: 10.1107/S1600536814005881/fy2112Isup2.hkl

Supporting information file. DOI: 10.1107/S1600536814005881/fy2112Isup3.mol

Supporting information file. DOI: 10.1107/S1600536814005881/fy2112Isup4.cml

CCDC reference: 992181

Additional supporting information:  crystallographic information; 3D view; checkCIF report

## Figures and Tables

**Table 1:** Hydrogen-bond geometry (Å, °)

*D*—H⋯*A*	*D*—H	H⋯*A*	*D*⋯*A*	*D*—H⋯*A*
C10—H10⋯O1^i^	0.93	2.82	3.422 (2)	124
C15—H15⋯O1^ii^	0.93	2.69	3.477 (2)	142

Symmetry codes: (i) 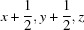; (ii) 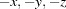.

**Fig. 1.**
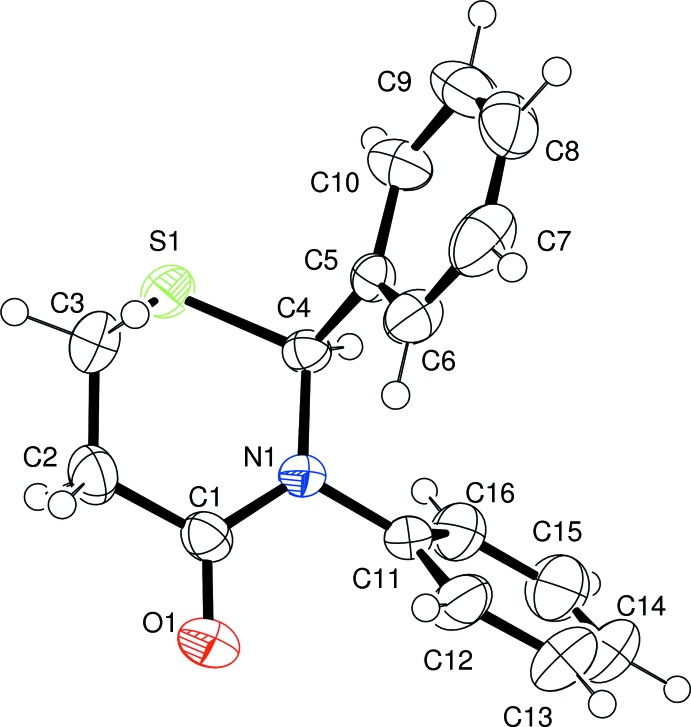
ORTEP view of the title comound. Thermal ellipsoids are drawn at 50% probability.

**Fig. 2.**
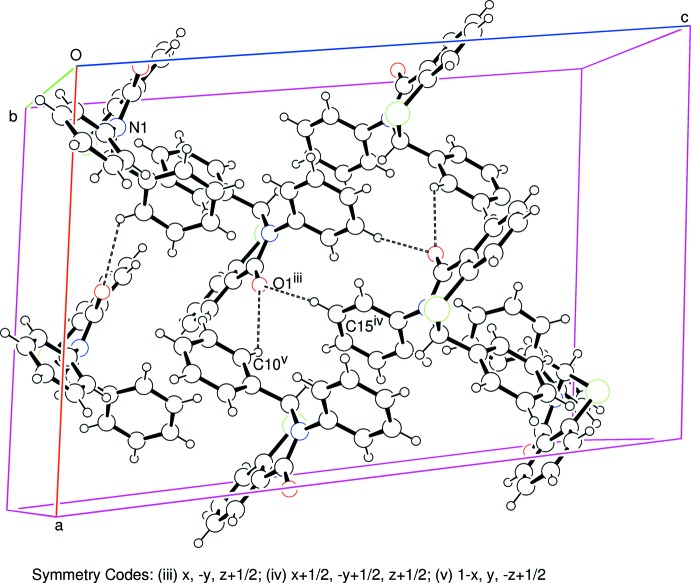
Crystal packing. C—H···O interactions are shown as dashed lines.

## supplementary crystallographic information

### 1. Comment

We report here the crystal structure (Fig. 1) of the title compound, which has two phenyl rings connected to the central thiazine ring and a third phenyl ring fused to the thiazine ring. We have recently reported the synthesis and crystal structures of three related compounds: (i) 2-(3-nitrophenyl)-3-phenyl-2,3-dihydro-4*H*-1,3-benzothiazin-4-one (Yennawar, Silverberg, Minehan and Tierney, 2013) (ii) 2,3-diphenyl-2,3,5,6-tetrahydro-4*H*-1,3-thiazin-4-one (Yennawar & Silverberg, 2014) and (iii) 6,7-diphenyl-5-thia-7-azaspiro[2.6]nonan-8-one (Yennawar & Silverberg, 2013). In order to more directly compare the structure of the benzothiazinone ring to the thiazinone and thiazepanone, we have obtained the crystal structure of the title compound, which, like the latter two compounds does not have a substituent on the *C*-phenyl ring. In the present structure, the dihedral angle between the phenyl rings is 74.25 (6)° - almost perpendicular and within the range of 60–90° in the three other compounds we have reported (see above). The thiazine ring has an envelope conformation with the 2-carbon forming the flap. In the crystal packing (Fig 2), molecules are linked by weak C—H···O interactions (Table 1).

The title compound has been synthesized previously (Ponci *et al.*, 1963; Kollenz & Ziegler, 1970; Oae & Numata, 1974; Badea *et al.*, 1998), but not by condensation of *N*-benzylideneaniline with thiosalicylic acid. In our hands, this reaction was not successfully accomplished in refluxing toluene or xylenes, with sodium sulfate in dioxane (Kamel *et al.*, 2010), with *p*-toluenesulfonic acid in refluxing toluene (Zarghi *et al.*, 2009), or with *N*,*N*'-dicyclohexylcarbodiimide (DCC) in THF (Zhou *et al.*, 2008; Srivastava *et al.*, 2002). The title molecule was finally synthesized by condensation in the presence of 2,4,6-tripropyl-1,3,5,2,4,6-trioxatriphosphorinane-2,4,6-trioxide (T3P) and pyridine, as per our previous reports.

### 2. Experimental

A two-necked 25 ml roundbottom flask was oven-dried, cooled under N_2_, and charged with a stir bar and *N*-benzylideneaniline (1.02 g, 6 mmol). Tetrahydrofuran (2.3 ml) was added, the solid dissolved, and the solution was stirred. Pyridine (1.95 ml, 24 mmol) was added and then thiosalicylic acid (0.931 g, 6 mmol) was added. Finally, 2,4,6-tripropyl-1,3,5,2,4,6-trioxatriphosphorinane-2,4,6-trioxatriphosphorinane-2,4,6-trioxide in 2-methyltetrahydrofuran (50 weight percent; 7.1 ml, 12 mmol) was added. The reaction was stirred at room temperature for 21 h, then poured into a separatory funnel and extracted three times with ethyl acetate. The organic was washed with saturated sodium bicarbonate, water and saturated sodium chloride. The solution was concentrated *in vacuo*, and then the solid was slurried in hot hexanes. After cooling in ice, the solid was collected by vacuum filtration and rinsed with cold hexanes to give a light orange solid (0.8697 g, m.p. 129–133°C). The solid was then recrystallized from ethanol, yielding solid that was still impure by TLC and melting point [0.5921 g, m.p. 132–134°C (lit. 138–140°C; Ponci *et al.*, 1963)]. R_f_ = 0.33 (20% EtOAc/hexanes). Crystals for X-ray crystallography were grown by slow evaporation from toluene.

### 3. Refinement

The C-bound H atoms were geometrically placed with C—H = 0.93–0.97 Å, and refined as riding with *U*_iso_(H) = 1.2 U_eq_(C).

### Crystal data

**Table:**

C_20_H_15_NOS	*F*(000) = 1328
*M**_r_* = 317.39	*D*_x_ = 1.334 Mg m^−^^3^
Monoclinic, *C*2/*c*	Mo *K*α radiation, λ = 0.71073 Å
*a* = 14.799 (4) Å	Cell parameters from 4538 reflections
*b* = 9.606 (3) Å	θ = 2.5–28.0°
*c* = 22.492 (6) Å	µ = 0.21 mm^−^^1^
β = 98.736 (5)°	*T* = 298 K
*V* = 3160.1 (15) Å^3^	Block, colourless
*Z* = 8	0.18 × 0.16 × 0.05 mm

### Data collection

**Table:**

Bruker SMART APEX CCD area-detector diffractometer	3956 independent reflections
Radiation source: fine-focus sealed tube	3300 reflections with *I* > 2σ(*I*)
Graphite monochromator	*R*_int_ = 0.021
Detector resolution: 8.34 pixels mm^-1^	θ_max_ = 28.4°, θ_min_ = 1.8°
φ and ω scans	*h* = −18→19
Absorption correction: multi-scan (*SADABS*; Bruker, 2001)	*k* = −12→12
*T*_min_ = 0.963, *T*_max_ = 0.990	*l* = −30→30
14747 measured reflections	

### Refinement

**Table:**

Refinement on *F*^2^	Primary atom site location: structure-invariant direct methods
Least-squares matrix: full	Secondary atom site location: difference Fourier map
*R*[*F*^2^ > 2σ(*F*^2^)] = 0.051	Hydrogen site location: inferred from neighbouring sites
*wR*(*F*^2^) = 0.127	H-atom parameters not refined
*S* = 1.06	*w* = 1/[σ^2^(*F*_o_^2^) + (0.0565*P*)^2^ + 1.8423*P*] where *P* = (*F*_o_^2^ + 2*F*_c_^2^)/3
3956 reflections	(Δ/σ)_max_ < 0.001
208 parameters	Δρ_max_ = 0.28 e Å^−^^3^
0 restraints	Δρ_min_ = −0.25 e Å^−^^3^

### Special details

**Table:**

Geometry. All e.s.d.'s (except the e.s.d. in the dihedral angle between two l.s. planes) are estimated using the full covariance matrix. The cell e.s.d.'s are taken into account individually in the estimation of e.s.d.'s in distances, angles and torsion angles; correlations between e.s.d.'s in cell parameters are only used when they are defined by crystal symmetry. An approximate (isotropic) treatment of cell e.s.d.'s is used for estimating e.s.d.'s involving l.s. planes.
Refinement. Refinement of *F*^2^ against ALL reflections. The weighted *R*-factor *wR* and goodness of fit *S* are based on *F*^2^, conventional *R*-factors *R* are based on *F*, with *F* set to zero for negative *F*^2^. The threshold expression of *F*^2^ > σ(*F*^2^) is used only for calculating *R*-factors(gt) *etc*. and is not relevant to the choice of reflections for refinement. *R*-factors based on *F*^2^ are statistically about twice as large as those based on *F*, and *R*- factors based on ALL data will be even larger.

### Fractional atomic coordinates and isotropic or equivalent isotropic displacement parameters (Å^2^)

**Table:**

	*x*	*y*	*z*	*U*_iso_*/*U*_eq_	
C1	0.03935 (11)	0.32561 (17)	0.11547 (7)	0.0434 (4)	
C2	0.01411 (11)	0.46594 (17)	0.13594 (7)	0.0434 (4)	
C3	0.04910 (12)	0.58978 (17)	0.11600 (7)	0.0460 (4)	
C4	0.18665 (11)	0.42745 (15)	0.09649 (7)	0.0406 (3)	
H4	0.2301	0.4022	0.0695	0.049*	
C5	0.24251 (10)	0.45006 (15)	0.15831 (7)	0.0377 (3)	
C6	0.23025 (12)	0.37177 (17)	0.20814 (8)	0.0469 (4)	
H6	0.1848	0.3041	0.2048	0.056*	
C7	0.28512 (13)	0.3933 (2)	0.26298 (8)	0.0557 (4)	
H7	0.2759	0.3408	0.2963	0.067*	
C8	0.35351 (13)	0.4925 (2)	0.26842 (9)	0.0559 (4)	
H8	0.3903	0.5069	0.3052	0.067*	
C9	0.36669 (12)	0.56958 (17)	0.21908 (9)	0.0519 (4)	
H9	0.4131	0.6357	0.2225	0.062*	
C10	0.31166 (11)	0.54979 (16)	0.16442 (8)	0.0442 (4)	
H10	0.3208	0.6034	0.1314	0.053*	
C11	0.14103 (11)	0.18738 (16)	0.06409 (7)	0.0393 (3)	
C12	0.21265 (16)	0.1052 (2)	0.08852 (9)	0.0657 (6)	
H12	0.2492	0.1317	0.1241	0.079*	
C13	0.23063 (18)	−0.0177 (2)	0.06018 (10)	0.0759 (7)	
H13	0.2795	−0.0733	0.0767	0.091*	
C14	0.17703 (15)	−0.05742 (19)	0.00829 (9)	0.0592 (5)	
H14	0.1882	−0.1412	−0.0100	0.071*	
C15	0.10730 (15)	0.0256 (2)	−0.01663 (9)	0.0670 (6)	
H15	0.0715	−0.0005	−0.0525	0.080*	
C16	0.08922 (14)	0.1488 (2)	0.01100 (8)	0.0603 (5)	
H16	0.0417	0.2057	−0.0065	0.072*	
C17	−0.05168 (12)	0.4728 (2)	0.17424 (8)	0.0514 (4)	
H17	−0.0753	0.3909	0.1877	0.062*	
C18	−0.08209 (14)	0.5990 (2)	0.19226 (9)	0.0619 (5)	
H18	−0.1253	0.6023	0.2182	0.074*	
C19	−0.04820 (14)	0.7204 (2)	0.17166 (10)	0.0660 (6)	
H19	−0.0696	0.8056	0.1834	0.079*	
C20	0.01695 (14)	0.7175 (2)	0.13392 (9)	0.0583 (5)	
H20	0.0394	0.8002	0.1204	0.070*	
N1	0.12016 (9)	0.31448 (13)	0.09313 (6)	0.0408 (3)	
O1	−0.01007 (9)	0.22507 (14)	0.11818 (6)	0.0617 (4)	
S1	0.13067 (3)	0.58704 (5)	0.06672 (2)	0.05412 (16)	

### Atomic displacement parameters (Å^2^)

**Table:**

	*U*^11^	*U*^22^	*U*^33^	*U*^12^	*U*^13^	*U*^23^
C1	0.0430 (9)	0.0427 (8)	0.0451 (8)	−0.0043 (7)	0.0089 (7)	−0.0052 (7)
C2	0.0396 (8)	0.0446 (9)	0.0443 (8)	0.0026 (7)	0.0007 (6)	−0.0060 (7)
C3	0.0447 (9)	0.0441 (9)	0.0454 (8)	0.0056 (7)	−0.0049 (7)	−0.0001 (7)
C4	0.0427 (8)	0.0361 (7)	0.0449 (8)	−0.0027 (6)	0.0124 (6)	0.0006 (6)
C5	0.0369 (8)	0.0311 (7)	0.0464 (8)	0.0018 (6)	0.0104 (6)	−0.0028 (6)
C6	0.0459 (9)	0.0435 (8)	0.0521 (9)	−0.0045 (7)	0.0107 (7)	0.0021 (7)
C7	0.0616 (11)	0.0576 (11)	0.0474 (9)	0.0031 (9)	0.0071 (8)	0.0055 (8)
C8	0.0561 (11)	0.0523 (10)	0.0555 (10)	0.0068 (9)	−0.0038 (8)	−0.0108 (8)
C9	0.0457 (9)	0.0382 (8)	0.0700 (11)	−0.0023 (7)	0.0033 (8)	−0.0101 (8)
C10	0.0457 (9)	0.0332 (7)	0.0552 (9)	−0.0018 (6)	0.0119 (7)	−0.0009 (7)
C11	0.0436 (8)	0.0360 (7)	0.0396 (7)	−0.0032 (6)	0.0111 (6)	−0.0018 (6)
C12	0.0748 (14)	0.0642 (12)	0.0528 (10)	0.0207 (10)	−0.0070 (9)	−0.0110 (9)
C13	0.1004 (18)	0.0631 (13)	0.0624 (12)	0.0353 (12)	0.0069 (12)	0.0007 (10)
C14	0.0841 (14)	0.0400 (9)	0.0607 (11)	−0.0032 (9)	0.0341 (10)	−0.0072 (8)
C15	0.0679 (13)	0.0740 (14)	0.0582 (11)	−0.0013 (11)	0.0070 (9)	−0.0278 (10)
C16	0.0606 (11)	0.0658 (12)	0.0513 (10)	0.0133 (10)	−0.0021 (8)	−0.0166 (9)
C17	0.0438 (9)	0.0568 (10)	0.0531 (9)	0.0044 (8)	0.0058 (7)	−0.0069 (8)
C18	0.0518 (11)	0.0707 (13)	0.0622 (11)	0.0164 (9)	0.0056 (9)	−0.0137 (10)
C19	0.0635 (12)	0.0582 (11)	0.0713 (12)	0.0262 (10)	−0.0057 (10)	−0.0156 (10)
C20	0.0636 (12)	0.0425 (9)	0.0630 (11)	0.0104 (8)	−0.0094 (9)	−0.0011 (8)
N1	0.0424 (7)	0.0363 (6)	0.0447 (7)	−0.0045 (5)	0.0101 (5)	−0.0051 (5)
O1	0.0595 (8)	0.0514 (7)	0.0800 (9)	−0.0190 (6)	0.0296 (7)	−0.0166 (6)
S1	0.0616 (3)	0.0450 (2)	0.0554 (3)	−0.0003 (2)	0.0080 (2)	0.01415 (18)

### Geometric parameters (Å, º)

**Table:**

C1—O1	1.218 (2)	C10—H10	0.9300
C1—N1	1.370 (2)	C11—C12	1.368 (2)
C1—C2	1.490 (2)	C11—C16	1.368 (2)
C2—C17	1.396 (2)	C11—N1	1.4401 (19)
C2—C3	1.398 (2)	C12—C13	1.386 (3)
C3—C20	1.397 (2)	C12—H12	0.9300
C3—S1	1.7587 (19)	C13—C14	1.362 (3)
C4—N1	1.4590 (19)	C13—H13	0.9300
C4—C5	1.521 (2)	C14—C15	1.356 (3)
C4—S1	1.8211 (16)	C14—H14	0.9300
C4—H4	0.9800	C15—C16	1.382 (3)
C5—C6	1.384 (2)	C15—H15	0.9300
C5—C10	1.393 (2)	C16—H16	0.9300
C6—C7	1.385 (2)	C17—C18	1.376 (3)
C6—H6	0.9300	C17—H17	0.9300
C7—C8	1.382 (3)	C18—C19	1.377 (3)
C7—H7	0.9300	C18—H18	0.9300
C8—C9	1.373 (3)	C19—C20	1.378 (3)
C8—H8	0.9300	C19—H19	0.9300
C9—C10	1.381 (2)	C20—H20	0.9300
C9—H9	0.9300		
			
O1—C1—N1	121.37 (15)	C12—C11—N1	120.90 (15)
O1—C1—C2	121.40 (15)	C16—C11—N1	119.66 (15)
N1—C1—C2	117.23 (14)	C11—C12—C13	119.86 (18)
C17—C2—C3	119.01 (16)	C11—C12—H12	120.1
C17—C2—C1	117.65 (15)	C13—C12—H12	120.1
C3—C2—C1	123.24 (15)	C14—C13—C12	120.3 (2)
C20—C3—C2	119.70 (17)	C14—C13—H13	119.8
C20—C3—S1	119.44 (14)	C12—C13—H13	119.8
C2—C3—S1	120.83 (13)	C15—C14—C13	119.79 (18)
N1—C4—C5	114.97 (12)	C15—C14—H14	120.1
N1—C4—S1	109.96 (11)	C13—C14—H14	120.1
C5—C4—S1	111.69 (10)	C14—C15—C16	120.32 (18)
N1—C4—H4	106.6	C14—C15—H15	119.8
C5—C4—H4	106.6	C16—C15—H15	119.8
S1—C4—H4	106.6	C11—C16—C15	120.21 (18)
C6—C5—C10	118.58 (15)	C11—C16—H16	119.9
C6—C5—C4	122.85 (14)	C15—C16—H16	119.9
C10—C5—C4	118.51 (14)	C18—C17—C2	120.88 (19)
C5—C6—C7	120.57 (16)	C18—C17—H17	119.6
C5—C6—H6	119.7	C2—C17—H17	119.6
C7—C6—H6	119.7	C17—C18—C19	119.67 (19)
C8—C7—C6	120.27 (17)	C17—C18—H18	120.2
C8—C7—H7	119.9	C19—C18—H18	120.2
C6—C7—H7	119.9	C18—C19—C20	120.95 (18)
C9—C8—C7	119.51 (17)	C18—C19—H19	119.5
C9—C8—H8	120.2	C20—C19—H19	119.5
C7—C8—H8	120.2	C19—C20—C3	119.77 (19)
C8—C9—C10	120.57 (17)	C19—C20—H20	120.1
C8—C9—H9	119.7	C3—C20—H20	120.1
C10—C9—H9	119.7	C1—N1—C11	119.46 (13)
C9—C10—C5	120.50 (16)	C1—N1—C4	122.85 (13)
C9—C10—H10	119.8	C11—N1—C4	117.68 (12)
C5—C10—H10	119.8	C3—S1—C4	95.64 (7)
C12—C11—C16	119.43 (16)		
			
O1—C1—C2—C17	18.8 (2)	N1—C11—C16—C15	−178.59 (18)
N1—C1—C2—C17	−161.54 (14)	C14—C15—C16—C11	−0.6 (3)
O1—C1—C2—C3	−157.41 (17)	C3—C2—C17—C18	0.1 (3)
N1—C1—C2—C3	22.2 (2)	C1—C2—C17—C18	−176.35 (16)
C17—C2—C3—C20	−0.9 (2)	C2—C17—C18—C19	0.9 (3)
C1—C2—C3—C20	175.31 (15)	C17—C18—C19—C20	−1.0 (3)
C17—C2—C3—S1	−178.93 (12)	C18—C19—C20—C3	0.2 (3)
C1—C2—C3—S1	−2.7 (2)	C2—C3—C20—C19	0.8 (3)
N1—C4—C5—C6	−1.3 (2)	S1—C3—C20—C19	178.82 (14)
S1—C4—C5—C6	124.86 (14)	O1—C1—N1—C11	9.4 (2)
N1—C4—C5—C10	175.80 (13)	C2—C1—N1—C11	−170.25 (13)
S1—C4—C5—C10	−58.01 (16)	O1—C1—N1—C4	−171.62 (16)
C10—C5—C6—C7	0.6 (2)	C2—C1—N1—C4	8.8 (2)
C4—C5—C6—C7	177.72 (15)	C12—C11—N1—C1	−114.62 (19)
C5—C6—C7—C8	−0.7 (3)	C16—C11—N1—C1	66.1 (2)
C6—C7—C8—C9	0.0 (3)	C12—C11—N1—C4	66.3 (2)
C7—C8—C9—C10	0.7 (3)	C16—C11—N1—C4	−113.00 (18)
C8—C9—C10—C5	−0.7 (3)	C5—C4—N1—C1	75.41 (18)
C6—C5—C10—C9	0.1 (2)	S1—C4—N1—C1	−51.67 (17)
C4—C5—C10—C9	−177.16 (14)	C5—C4—N1—C11	−105.55 (15)
C16—C11—C12—C13	−1.6 (3)	S1—C4—N1—C11	127.37 (12)
N1—C11—C12—C13	179.11 (19)	C20—C3—S1—C4	148.70 (14)
C11—C12—C13—C14	−0.4 (4)	C2—C3—S1—C4	−33.26 (15)
C12—C13—C14—C15	1.8 (4)	N1—C4—S1—C3	56.83 (12)
C13—C14—C15—C16	−1.3 (3)	C5—C4—S1—C3	−72.06 (12)
C12—C11—C16—C15	2.1 (3)		

### Hydrogen-bond geometry (Å, º)

**Table:**

*D*—H···*A*	*D*—H	H···*A*	*D*···*A*	*D*—H···*A*
C10—H10···O1^i^	0.93	2.82	3.422 (2)	124
C15—H15···O1^ii^	0.93	2.69	3.477 (2)	142

Symmetry codes: (i) *x*+1/2, *y*+1/2, *z*; (ii) −*x*, −*y*, −*z*.

## References

[bb1] Badea, F., Costea, I., Iordache, F., Simion, A. & Simion, C. (1998). *Rev. Roum. Chim.* **43**, 675–678.

[bb2] Bruker (2001). *SMART*, *SADABS* and *SAINT* Bruker AXS Inc., Madison, Wisconsin, USA.

[bb3] Farrugia, L. J. (2012). *J. Appl. Cryst.* **45**, 849–854.

[bb4] Kamel, M. M., Ali, H. I., Anwar, M. M., Mohamed, N. A. & Soliman, A. M. M. (2010). *Eur. J. Med. Chem.* **45**, 572–580. 10.1016/j.ejmech.2009.10.044 19932530

[bb5] Kollenz, G. & Ziegler, E. (1970). *Monatsh. Chem.* **101**, 97–101.

[bb6] Oae, S. & Numata, T. (1974). *Tetrahedron*, **30**, 2641–2646.

[bb7] Ponci, R., Baruffini, A. & Gialdi, F. (1963). *Il Farmaco Ed. Sci.* **18**, 653–657. 14096614

[bb8] Sheldrick, G. M. (2008). *Acta Cryst.* A**64**, 112–122. 10.1107/S0108767307043930 18156677

[bb9] Srivastava, T., Haq, W. & Katti, S. B. (2002). *Tetrahedron*, **58**, 7619–7624.

[bb10] Yennawar, H. P. & Silverberg, L. J. (2013). *Acta Cryst.* E**69**, o1659. 10.1107/S1600536813027979 PMC3884320 24454096

[bb11] Yennawar, H. P. & Silverberg, L. J. (2014). *Acta Cryst.* E**70**, o133. 10.1107/S1600536814000324 PMC3998299 24764860

[bb12] Yennawar, H. P., Silverberg, L. J., Minehan, M. J. & Tierney, J. (2013). *Acta Cryst.* E**69**, o1679. 10.1107/S1600536813028389 PMC3884335 24454111

[bb13] Zarghi, A., Zebardast, T., Daraie, B. & Hedayati, M. (2009). *Bioorg. Med. Chem.* **17**, 5369–5373. 10.1016/j.bmc.2009.06.056 19596198

[bb14] Zhou, H., Liu, A., Li, X., Ma, X., Feng, W., Zhang, W. & Yan, B. (2008). *J. Comb. Chem.* **10**, 303–312. 10.1021/cc700164u 18163593

